# Acutely Inhibiting AQP4 With TGN-020 Improves Functional Outcome by Attenuating Edema and Peri-Infarct Astrogliosis After Cerebral Ischemia

**DOI:** 10.3389/fimmu.2022.870029

**Published:** 2022-05-03

**Authors:** Chengfeng Sun, Luyi Lin, Lekang Yin, Xiaozhu Hao, Jiaqi Tian, Xiaoxue Zhang, Yan Ren, Chanchan Li, Yanmei Yang

**Affiliations:** ^1^ Department of Radiology, Huashan Hospital, Fudan University, Shanghai, China; ^2^ Department of Radiology, Zhongshan Hospital, Fudan University, Shanghai, China; ^3^ Department of Radiology, Renji Hospital, Shanghai Jiao Tong University, Shanghai, China; ^4^ Department of Radiotherapy, Shanghai Eastern Hepatobiliary Surgery Hospital, Shanghai, China

**Keywords:** ischemic stroke, astrogliosis, AQP4 polarization, glymphatic system, ultra-high b-values diffusion weighted imaging

## Abstract

**Background:**

Ischemic stroke is one of the leading causes of human death and disability. Brain edema and peri-infarct astrocyte reactivity are crucial pathological changes, both involving aquaporin-4 (AQP4). Studies revealed that acute inhibition of AQP4 after stroke diminishes brain edema, however, its effect on peri-infarct astrocyte reactivity and the subacute outcome is unclear. And if diffusion-weighted imaging (DWI) could reflect the AQP4 expression patterns is uncertain.

**Methods:**

Rats were subjected to middle cerebral artery occlusion (MCAO) and allocated randomly to TGN 020-treated and control groups. One day after stroke, brain swelling and lesion volumes of the rats were checked using T2-weighted imaging (T2-WI). Fourteen days after stroke, the rats successively underwent neurological examination, T2-WI and DWI with standard b-values and ultra-high b-values, apparent diffusion coefficient (ADC) was calculated correspondingly. Finally, the rats’ brains were acquired and used for glial fibrillary acidic protein (GFAP) and AQP4 immunoreactive analysis.

**Results:**

At 1 day after stroke, the TGN-020-treated animals exhibited reduced brain swelling and lesion volumes compared with those in the control group. At 14 days after stroke, the TGN-020-treated animals showed fewer neurological function deficits and smaller lesion volumes. In the peri-infarct region, the control group showed evident astrogliosis and AQP4 depolarization, which were reduced significantly in the TGN-020 group. In addition, the ultra-high b-values of ADC (ADCuh) in the peri-infarct region of the TGN-020 group was higher than that of the control group. Furthermore, correlation analysis revealed that peri-infarct AQP4 polarization correlated negatively with astrogliosis extent, and ADCuh correlated positively with AQP4 polarization.

**Conclusion:**

We found that acutely inhibiting AQP4 using TGN-020 promoted neurological recovery by diminishing brain edema at the early stage and attenuating peri-infarct astrogliosis and AQP4 depolarization at the subacute stage after stroke. Moreover, ADCuh could reflect the AQP4 polarization.

## Introduction

Ischemic stroke is a leading cause of death and disability in humans, with few pathophysiological therapies other than recanalizing occluded blood vessels (1, 2). Acutely inhibiting aquaporin-4 (AQP4) was proposed recently as a promising new pathophysiological therapy targeting central nervous system (CNS) edema post-injury (3, 4). Because water transportation through AQP4 is a passive process, depending on osmotic gradients. AQP4 contributes to the formation of cellular toxic edema at first, but it is also essential for the resolution of vasogenic edema in CNS injury. And studies revealed that AQP4 deficient animals displayed higher levels of CNS water content than control animals at a later phase of CNS injury (5). AQP4 is the most abundant aquaporins in the brain, it has a polarized distribution tendency on the astrocyte endfeet facing vessels under physiological conditions, this distribution tendency is critical for the formation and resolution of edema, and clearance of interstitial solutes in the brain (6). Commonly, methods of inhibiting AQP4 mainly include gene knockout, small interfering RNA, heavy metal ions, and small molecule inhibitors (7). Small molecule inhibitors have the potential to be applied in clinical for their security. N-(1,3,4-thiadiazol-2-yl) pyridine-3-carboxamide dihydrochloride (TGN-020) is one of them and has been proven to inhibit AQP4 *in vitro* and *in vivo via* the intracellular ubiquitin-proteasome system (8, 9).

AQP4 is implicated not only in edema formation and resolution, but also in astrocyte migration and astrogliosis (10, 11). However, the changes in peri-infarct astrocyte reactivity related to acute inhibition of AQP4 have not been clarified, which are crucial for peri-infarct tissue repair and neurological function recovery. After stroke, reactive astrogliosis and loss of perivascular AQP4 polarization occur and persist for long time in the peri-infarct area (12–14). Reactive astrogliosis is beneficial for limiting the infarct territory initially; however, its increasing dysregulation at the recovery stage accentuates inflammation and inhibits axon regeneration, thus interfering with long-term sensorimotor functional recovery (15, 16). Besides, loss of AQP4 polarization impairs the glymphatic system, a newly-discovered waste clearance system in the brain (17), causing toxic protein deposition and cognitive deficits (18, 19). Modulating reactive astrogliosis and the loss of AQP4 polarization in the peri-infarct area might be beneficial therapeutic strategies during later stages to promote neurological function recovery.

In this study, we acutely inhibited AQP4 using TGN-020 in transient middle cerebral artery occlusion (MCAO) rats, evaluated the brain edema and infarct volume at 1 and 14 days, and the peri-infarct astrogliosis extent, AQP4 expression patterns, and neurological function at 14 days after MCAO. In addition, we analyzed correlations of the AQP4 expression patterns and the ultra-high b-values apparent diffusion coefficient (ADCuh). We aimed to investigate the effect of acutely inhibiting AQP4 on peri-infarct astrocyte reactivity and subacute outcome and the feasibility of ADC to reflect the expression patterns of AQP4.

## Material and Methods

### Animals

This experiment was approved by the Fudan University Institutional Animal Care and Use Committee. A total of 16 adult (260–280 g) Sprague–Dawley rats (Charles River Laboratories, Beijing, China) were used in this experiment. They were maintained under an automatically controlled 12 h light–dark cycle, with freely accessible food and water. After fasting for 1 day, the rats were subjected to 90 min of MCAO and then allocated randomly to the TGN-020 treated group or the control group (n = 8 per group). The ischemic lesion and edema volume were checked by MRI 1 d post-stroke. At 14 days post-stroke, neurological function, MRI, and histology features were evaluated in turn. One rat in the TGN-020-treated group and three rats in the control group died from severe ischemic stroke. Finally, six rats of each group were included in the data analysis.

### Surgical Procedure and Treatment

For all rats, the left middle cerebral artery was occluded by the same researcher as in our previous study (20). Specifically, the anesthetized rats were immobilized in a supine position using a tooth holder and all limbs were tied up. A skin incision was made in the midline of the neck, and the muscle and fascia were separated to expose the left internal carotid artery (ICA), external carotid artery, and common carotid artery. Then, a poly L-lysine coated nylon filament (2634A4, Cinontech Co. Ltd., Beijing, China) was inserted into the left ICA to block blood flow to the MCA. The TGN-020 treated group was administrated intraperitoneally with TGN-020 (200 mg/kg) at 10 minutes after successful occlusion. The control group was given the same volume of 0.9% normal saline at the same timepoint. After occlusion for 90 minutes, the filament was withdrawn gently to allow reperfusion under anesthesia.

### MRI and Quantitative Analysis

The MRI images were captured using a 3.0T horizontal magnet (Discovery MR750, GE Medical Systems, Milwaukee, WI, USA) with a 60-mm-diameter gradient coil (Magtron Inc., Jiangyin, China). Anesthetized rats were scanned in the prone position, with continuous monitoring of their temperature, heart rate, and respiration. The main scan parameters were as follows: For fast spin echo T2-weighted imaging, repetition time (TR)/echo time (TE) = 4000 ms/96 ms, field of view (FOV) = 6 cm × 6 cm, matrix = 256 × 256, slice thickness = 1.8 mm, interslice distance = 2 mm, number of slices = 15. For ultra-high diffusion-weighted imaging (DWIuh), TR/TE = 3000/minimum, FOV = 6 cm × 6 cm, slice thickness = 1.8 mm, interslice distance = 2 mm, number of slices = 15, b values = 2000, 2500, 3000, 3500, 4000, and 4500 s/mm^2^. Standard DWI (DWIst) was performed with the same parameters as DWIuh, except that the b values = 0, 800 s/mm^2^. T2-WI was scanned at 1 day and 14 days post-stroke, while DWIst and DWIuh were scanned at 14 days post-stroke.

All MRI data were processed and measured on the GE ADW4.6 workstation using Functool software, and DWI images were processed to generate ADC maps. The ischemic lesion volume was calculated as percentage of hemispheric lesion volume (%HLV) after correction of hemispheric space-occupying effects, according to methods proposed by Gerriets et al. (21). The percentage of brain swelling volume (%BSV) was used to quantitatively evaluate the extent of brain swelling. The specific equations used are as follows:


%HLV={[contralateral hemisphere volume−(ipsilateral hemisphere volume−infarct volume)]/contralateral hemisphere volume} ×100

%BSV= (ipsilateral hemisphere volume/contralateral hemisphere volume)×100.

Imaging artifacts increase when the b-values rise, especially in the cortical region, thus the estimation of ADCuh was only carried out in the peri-infarct striatum. Equivalent regions of interest (ROIs) were drawn in the peri-infarct striatum and corresponding contralateral area on ADCuh maps. The ratio of ipsilateral ADCuh to contralateral ADCuh was used for comparisons between groups.

### Sensorimotor and Cognitive Function Examination

A neurological behavior scale of 0 to 20 scores was used to assess the sensorimotor function of the rats, as in our previous study (22). Higher scores represent more neurological deficits. The Y-maze was used to test the spatial working memory of the rats, based on the inherent characterization of rats to explore a novel environment without the need to learn skills. The maze is consisted of three identical arms (50 cm × 16 cm × 32 cm), and the angle between each arm was 120°. Rats were placed at the end of the initial arm and were allowed to explore freely for 5 minutes. The sequence and total number of arm entries were recorded using a video camera. Entrance into different arms for three consecutive times was recorded as a correct alternating response. Rodents with impaired working memory could not memorize which arm was just visited and thus had lower spontaneous alternation rates. Correct alternating response times were counted, and the spontaneous alternation rate was calculated using the following equation:


Spontaneous alternation rate=[correct alternating response times/(N−2)]×100%, where N is the total number of arm entries.

### Immunofluorescence Staining and Quantitative Analysis

Rats were perfused with phosphate buffer, followed by 4% paraformaldehyde, and then their brains were removed and postfixed overnight at 4°C. After dehydration, wax leaching, embedding, and slicing, three serial coronary brain sections (thickness: 5 μm) for each animal were obtained at approximately 0.24 mm relative to the bregma, according to the atlas reported by Paxinos and Watson (2005). Well preserved sections were picked and immunostained using anti-glial fibrillary acidic protein (GFAP, 1:1000, Abcam, Cambridge, MA, USA) and anti-AQP4 (1:1000, Abcam) antibodies. Alexa Fluor 488- and 568-conjugated donkey anti-rabbit and anti-mouse antibodies (1:1000, Abcam) were used as secondary antibodies. Finally, the sections were incubated with 4′,6-diamidino-2-phenylindole, dihydrochloride (DAPI, 1: 1000; Sigma-Aldrich, St. Louis, MO, USA).

Immunofluorescence sections were scanned using a Vslide scanning microscope (Nikon, Chiyoda, Tokyo, Japan) with a ×20 primary objective. All images were acquired using constant scanning settings, and further semi-quantitatively analyzed to characterize the expression patterns of AQP4 and GFAP using Image J (National Institutes of Health, Bethesda, MD, USA).

To evaluate AQP4 expression and polarization in the peri-infarct area, the mean fluorescence intensity of AQP4 emission channels was measured, and AQP4 polarization was calculated as the ratio of the low-threshold AQP4-positive area to the high-threshold AQP4 positive area (23). The percentage of GFAP immunostained area of the ROIs (GFAP area%) was used to analyze reactive astrogliosis. ROIs (600 μm × 300 μm) were placed in the peri-infarct cortex and striatum separately for analysis. Immunostained sections that had similar lesion morphologies and anatomical structures to those in the ADCuh images were picked for analysis, and ROIs in the peri-infarct striatum were placed according to those ROIs placed in the ADCuh images. All histological data were normalized by contralateral values and were calculated twice to minimize measurement error.

### Statistical Analysis

All data were presented as the mean ± the standard deviation (SD), *P* < 0.05 was considered to be statistically significant. One-way analysis of variance (ANOVA) and *post hoc* least significant difference (LSD) tests were used to compare differences among groups. Pearson Product correlation analysis was performed to analyze correlations. The above data analyses were carried out using GraphPad Prism, version 8.0 (GraphPad Software Inc., La Jolla, CA, USA).

## Results

### T2-WI Revealed That Acute Inhibition of AQP4 Decreased Edema and the Infarct Volume

**Figure 1:**
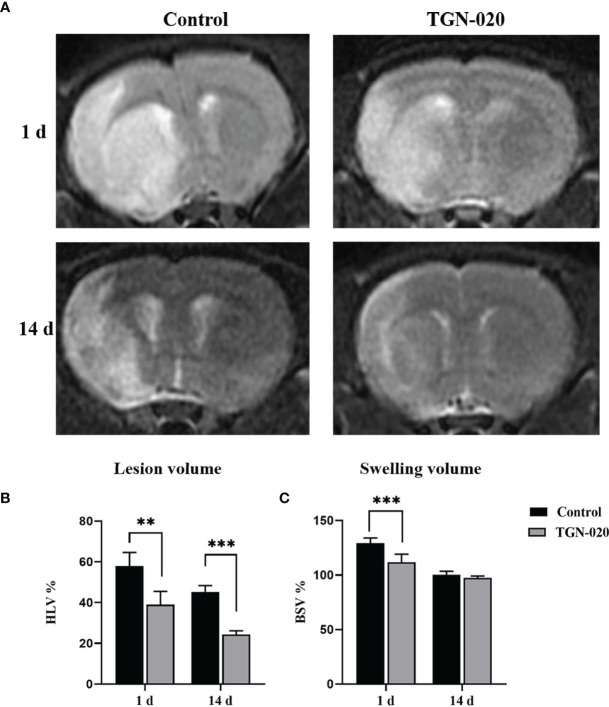
Comparison of ischemic lesion volumes and brain swelling volumes between groups. **(A)** Representative T2-WI images of rats in the TGN-020-treated and control groups at 1 day and 14 days post-stroke. **(B)** The ischemic Lesion volumes of each group at 1 day and 14 days post-stroke. **(C)** The brain swelling volumes of each group at 1 day and 14 days post-stroke. **P < 0.01, ***P < 0.001.

Ischemic lesion volume and brain swelling extent of the rats were derived from T2-WI at 1 day and 14 days post stroke (**Figure 1**). One day post stroke, the TGN-020-treated group presented significantly decreased infarct and swelling volumes (%HLV: 39.05 ± 6.43, %BSV: 111.98 ± 7.18), compared with those of the control group (%HLV: 57.94 ± 6.68, %BSV: 129.32 ± 4.69). Fourteen days later, the ischemic lesion volume and brain swelling extent of both groups had decreased. The TGN-020-treated group had a smaller infarct volume (%HLV: 24.30 ± 1.88) than that of control group (%HLV: 45.25 ± 3.11). Regarding the extent of brain swelling, no significant difference was found between two groups. Our results showed a 67% smaller lesion volume with 86% less swelling in TGN-020-treated rats compared with those of the control rats at 1 day-post stroke (both *P* < 0.01), and a 53% smaller lesion volume at 14 days (*P* < 0.001).

### Acute Inhibition of AQP4 Ameliorated Neurological Deficits

**Figure 2:**
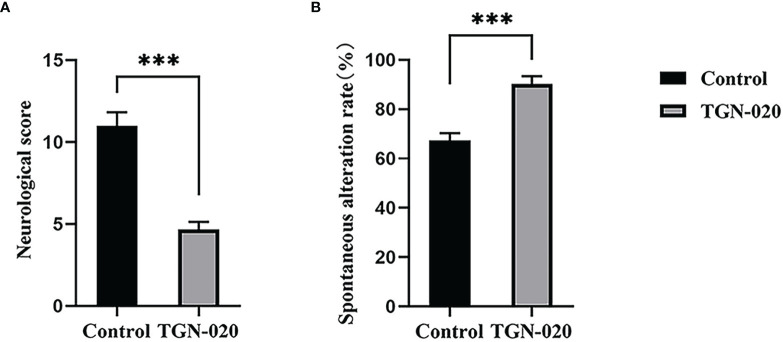
TGN-020-treated rats showed improved neurological function. **(A)** Comparison of neurological scores between groups revealed fewer sensorimotor deficits in the TGN-020 treated rats compared with those in the control rats. **(B)** Comparison of spatial working memory by spontaneous alternation in the Y maze showed that the TGN-020 treated rats had better cognitive function than the control rats. ***P < 0.001.

At fourteen days post stroke, the sensorimotor function of the rats was evaluated using the behavior scale. Significantly fewer sensorimotor deficits were observed in the TGN-020-treated group (*P* < 0.001 *vs*. the control group). Spatial working memory was assessed using Y maze spontaneous alternation, in which the TGN-020-treated group showed a superior cognition performance compared with that of the control group (*P* < 0.001) (**Figure 2**).

### Acutely Inhibiting AQP4 Ameliorated Peri-Infarct Astrogliosis and Loss of AQP4 Polarization

**Figure 3:**
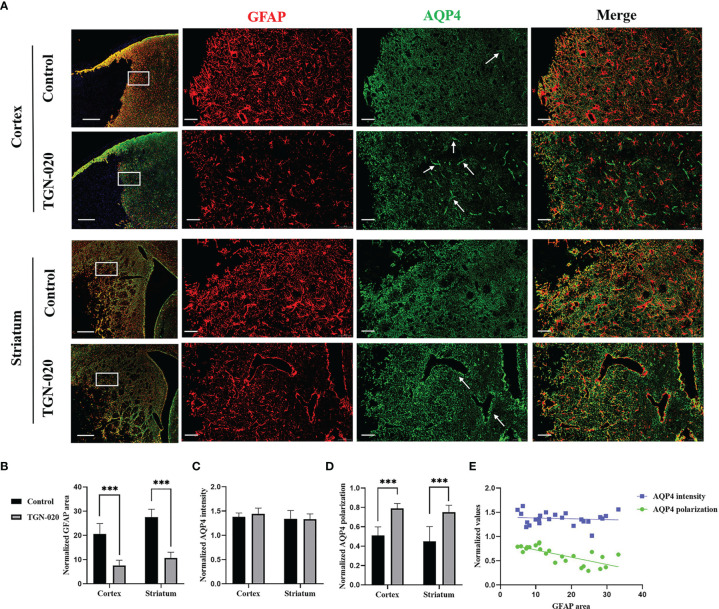
Peri-infarct astrogliosis and AQP4 expression patterns in the two groups of rats. **(A)** Immunostaining of GFAP (red) and AQP4 (green) in the peri-infarct cortex and striatum in TGN-020- treated rats and control rats. White boxes in the first column indicate the ROIs used for GFAP and AQP4 analysis. The arrows show AQP4 located in the perivascular region. Scale bars = 500 and 50 µm. **(B–D)** Comparisons of GFAP-positive area and AQP4 expression patterns in the peri-infarct cortex and striatum between the two groups. **(E)** Correlations between peri-infarct AQP4 expression patterns and the extent of astrogliosis. ***P < 0.001.

In the peri-infarct cortex and striatum, the TGN-020-treated group showed fewer and smaller astrocytes than those in the control group. AQP4 in the control group was located diffusely on the neuropil, while AQP4 in the TGN-020-treated group was distributed mainly in the perivascular district, which is close to the polarized distribution under normal conditions. Corresponding immunofluorescence images of each group are shown in **Figure 3**. Quantitively, in the peri-infarct area, the TGN-020-treated group exhibited smaller cortical and striatal GFAP area (7.57 ± 2.18 and 10.72 ± 2.32, respectively) than those of the control group (both *P* < 0.001). The AQP4 expression intensity (the AQP4 mean fluorescence intensity) of the two groups were similar (*P* > 0.05). The cortical and striatal AQP4 polarizations of the TGN-020-treated group were higher than those of the control group (cortex: 0.78 ± 0.06 in TGN-020-treated group *vs*. 0.48 ± 0.09 in the control group, *P* < 0.01; striatum: AQP4 polarization: 0.75 ± 0.07 in TGN-020-treated group *vs*. 0.43 ± 0.15 in the control group, *P* < 0.001). Further correlation analysis showed that peri-infarct AQP4 polarization correlated negatively with the astrogliosis area (*r* = −0.72, *P* < 0.01).

### Relationship of the ADCuh With AQP4 Expression Patterns

**Figure 4:**
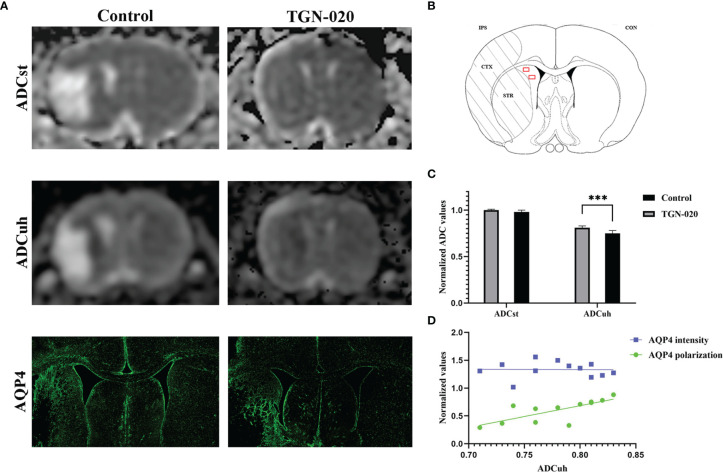
Correspondence between ADC and AQP4 expression patterns. **(A)** Representative ADCst and ADCuh maps, and AQP4 staining patterns in TGN-020-treated and control rats. **(B)** Anatomical reference showing the ROIs (red boxes) used to estimate the ADCuh in the peri-infarct striatum. **(C)** Comparison of ADCuh in the peri-infarct striatum between the two groups. **(D)** Correlations between the peri-infarct AQP4 expression patterns and ADCuh. ***P < 0.001. Con, contralateral; CTX, cortex; IPS, ipsilateral; STR, striatum.

On the standard ADC (ADCst) maps, there were no evident signal differences between the peri-infarct area and contralateral hemisphere in all rats, while on the ADCuh maps, the peri-infarct area appeared as dark rings surrounding the ischemic core in the two groups of rats (**Figure 4**). The ratio of the ipsilateral to contralateral ADC was used for group comparison, and was only analyzed in the striatum for restriction of ADCuh map’s artifacts. No significant difference in ADCst was found between the TGN-020-treated group and the control group (*P* > 0.05), while the TGN-020-treated group had a slightly but significantly increased ADCuh (0.78 ± 0.04) compared with that of the control group (0.73 ± 0.03, *P* < 0.05).

Both groups of rats showed reactive astrogliosis and loss of AQP4 polarization in the peri-infarct area, in which the ADCuh decreased concurrently (**Figure 4**). Further correlation analysis showed that the peri-infarct ADCuh correlated positively with AQP4 polarization (*r* = 0.64, *P* < 0.05), but had no statistical correlation with the AQP4 mean fluorescence intensity (*r* = 0.03, *P* = 0.92).

## Discussion

In the present study, acute inhibition of AQP4 using TGN-020 decreased the edema and infarct lesion volume 1day post-stroke, attenuated peri-infarct astrogliosis, AQP4 depolarization, and infarct lesion volume, promoting neurological recovery at 14 days post-stroke. Additionally, we found that AQP4 polarization correlated negatively with astrogliosis, and ADCuh could reflect the AQP4 polarization.

Our results showed that acute inhibition of AQP4 by TGN-020 reduced brain edema 1day post-stroke, which is consistent with previous research (24, 25). Traditionally, it is thought that post-stroke edema comprises cytotoxic edema and vasogenic edema, in which AQP4 plays inductive and counteractive roles, respectively, with edematous fluid mainly coming from blood plasma (26, 27). However, recently, researchers found that cerebrospinal fluid immediately flowed towards the brain parenchyma through the influx pathway of the glymphatic system after ischemic stroke, and an absence of AQP4 reduced the cerebrospinal fluid influx significantly (4, 28). Regardless, the traditional or newly-found mechanism of edema both suggest that acute inhibition of AQP4 could reduce post-stroke edema at the early stage (29, 30). However, perivascular AQP4 is essential for the dissipation of vasogenic edema and the glymphatic clearance of Aβ and tau (8, 9). The deficiency of AQP4 would cause the accumulation of water and neurotoxic protein in the recovery stage of CNS injury (3, 31). In this study, we further investigated the peri-infarct expression patterns of AQP4 14 days after acute inhibition of AQP4. No differences in AQP4 expression intensity were found between the TGN-020 group and the control group, but AQP4 polarization of the TGN-020 group was higher than that of the control group, in other words, the perivascular AQP4 was increased in the TGN-020 group when compared with the control group. As is shown in our study, swelling extent of the ipsilateral hemisphere has turned to normal in both groups 14 days post stroke. These AQP4 might not contribute to water transmembrane diffusivity, but play roles in neurotoxic waste elimination. Researchers found that toxic molecules present in the area of liquefactive necrosis can leak across the glial scar and were removed by the glymphatic system in peri-infarct tissue (32). So, it can be inferred that the higher AQP4 polarization of the TGN-020 group is beneficial for glymphatic clearance and neurological recovery.

For ischemic stroke and other multiple CNS diseases, peri-infarct reactive astrogliosis is usually accompanied by loss of AQP4 polarization in the same area (23, 33–35). Our experiment showed that the polarization of AQP4 correlated negatively with the astrogliosis area, indicating that the astrogliosis extent might affect the polarization of AQP4. The close relationship between astrogliosis and AQP4 polarization was also discovered in rodent models of traumatic brain injury and multiple microinfarcts, though needing further investigations to determine the underlying mechanisms. Some researchers regard the loss of AQP4 polarization as an important feature of reactive astrocytes rather than a pathological consequence of endfeet damage (34, 36). We consider that the decreased astrogliosis after acute inhibition of AQP4 might contribute to the preservation of AQP4 polarization.

The reactive astrogliosis that occurs after ischemic stroke is extremely complex and incompletely understood, playing both detrimental and beneficial roles on neurological recovery (37–39). Some studies found that reactive astrocyte was beneficial for vascular repair and axonal regrowth after CNS injury (40, 41), while other studies revealed that reactive astrocyte could restrict neural repair by expressing growth inhibitory factors and forming glial scars (42). These contradictory roles of reactive astrocytes may be due to different reactive phenotypes induced by injury (43, 44). The reactive astrocytes in neuroinflammation of ischemia could be classified into A1s and A2s, which exert different functions (45, 46). The A1s exert the neurotoxic role with classical complement cascade gene upregulation, while the A2s upregulate many neurotrophic factors to promote neuronal recovery (47, 48). Therapies aimed at enhancing pro-reparative functions and reducing harmful functions in reactive astrocytes may benefit the outcome of ischemic stroke (49).

In this study, acute inhibition of AQP4 reduced peri-infarct astrogliosis and preserved AQP4 polarization, accompanied by a decreased lesion volume and improved neurological function. AQP4 is implicated in astrocyte migration and astrogliosis after brain insult (50, 51), which was supported by the reduced peri-infarct astrogliosis after inhibition of AQP4 observed in our study. Moreover, we inferred that the reduced astrogliosis might attenuate inflammation and promote neural rejuvenation by reducing the number of neurotoxic A1s astrocytes, contributing to peri-infarct tissue repair and functional outcomes. Reactive astrocytes of different phenotypes exhibit double-edged effects on pathological progression (49, 52, 53), our experiments and substantive studies that demonstrated inhibiting reactive astrogliosis facilitated neural rejuvenation and the long-term functional outcome might be attributed to a decrease of the neurotoxic A1s astrocytes (54–56). Besides, we speculated that the preserved AQP4 polarization benefits the cognitive recovery of TGN-020-treated rats by increasing the drainage of toxic extracellular fluid in the core of the infarct. Perivascular AQP4 is a critical component of the brain glymphatic system (57, 58). The loss of AQP4 polarization would impair the clearance efficiency of the glymphatic system, resulting in toxic protein deposition and the induction of cognitive deficits after stroke (59, 60). Therapeutic strategies that improved the AQP4 polarization might be effective to enhance the glymphatic function and contribute to the neurological recovery (61).

Deciphering changes in AQP4 are helpful to understand its roles in the pathology of ischemic stroke; however, most analytical methods remain highly invasive or destructive. According to the literature, aquaporin overexpression produces contrast in DWI by increasing tissue water diffusivity (62). ADCuh (b values > 2000 s/mm^2^) could reflect the expression level of aquaporin by estimating water transmembrane diffusivity (63). However, the relationship between ADCuh and aquaporin expression patterns in different studies are controversial. Some studies found that ADCuh correlated positively with the aquaporins expression intensity in tumors (64, 65), however, studies on ischemic stroke showed that ADCuh correlated negatively with aquaporin expression intensity (66–68). In our study, ADCuh correlated positively with the polarization of AQP4 rather than its expression intensity. This was probably the result of no adequate deviations among the AQP4 expression intensity of rats in this study, which did not allow us to infer a statistically significant correlation with ADCuh. Besides, the polarization of AQP4 might be more consequential for the functions of the protein than its expression intensity, as implied by other studies (69, 70).

The present study had certain limitations. Firstly, the inconsistency of lesion volumes before intervention between groups was avoided to the greatest extent, however, it could still not be excluded from the analysis. Longitudinal studies including data before inhibiting AQP4 might be more conclusive. Secondly, because higher b-value images lead to more imaging artifacts, the correlations between ADCuh and AQP4 expression patterns were only analyzed in the peri-infarct striatum, thus further studies should be carried out using MRI machines with a higher performance. Thirdly, we didn’t use the gene transcriptome analysis or key molecular markers immunostaining to differentiate the specific changes of two groups of reactive astrocytes after acute inhibition of AQP4, which will be carried out in our further studies. Besides, we only evaluate the role of AQP4 inhibition after ischemia onset, without investigating the effect of AQP4 inhibition on astrocyte and venules after recirculation, giving the TGN-020 along with the removal of filament in establishing the artery occlusion stroke animal model may be helpful to answer that.

In conclusion, we found acutely inhibiting AQP4 with TGN-020 not only decreased the edema at the early stage of ischemic stroke but also reduced peri-infarct astrogliosis and AQP4 depolarization, promoting sensorimotor and cognitive recovery at the subacute stage. This study extends the evaluation timepoint of previous studies investigating the effect of TGN-020 on ischemic stroke, providing further supportive evidence that acute inhibition of AQP4 after stroke is a viable therapeutic strategy. Furthermore, we revealed that AQP4 polarization correlated negatively with astrogliosis in the peri-infarct area, indicating therapies targeting astrogliosis might be effective to preserve AQP4 polarization and promote neurological recovery in ischemic stroke. And our results showed that ADCuh could reflect the AQP4 expression patterns, it might be a useful tool to decipher the AQP4 expression noninvasively.

## Data Availability Statement

The raw data supporting the conclusions of this article will be made available by the authors, without undue reservation.

## Ethics Statement

The animal study was reviewed and approved by Fudan University Institutional Animal Care and Use Committee.

## Author Contributions

Study conception and design: YY and CS; experiment implementation, statistical analysis and figure preparation: CS, LL, XH, JT, and XZ; manuscript writing: CS and LY; paper reviewing: CL and YY. All authors read and approved the final manuscript.

## Funding

This research was supported by the National Natural Science Foundation of China, Nos. 81771788. The funding sources had no role in study conception and design, data analysis or interpretation, paper writing or deciding to submit this paper for publication.

## Conflict of Interest

The authors declare that the research was conducted in the absence of any commercial or financial relationships that could be construed as a potential conflict of interest.

## Publisher’s Note

All claims expressed in this article are solely those of the authors and do not necessarily represent those of their affiliated organizations, or those of the publisher, the editors and the reviewers. Any product that may be evaluated in this article, or claim that may be made by its manufacturer, is not guaranteed or endorsed by the publisher.
